# Placenta accreta after postpartum tubal sterilisation and Novasure^®^ endometrial ablation

**DOI:** 10.1002/ccr3.3962

**Published:** 2021-02-27

**Authors:** Nnadozie Igbokwe, Kevin Glackin, Harpreet Kaur

**Affiliations:** ^1^ Royal Jubilee Maternity Hospital Belfast UK; ^2^ Queens University Belfast Belfast UK; ^3^ Altnagelvin Area Hospital Londonderry UK; ^4^ Watford General Hospital Watford UK

**Keywords:** contraception, endometrial ablation, placenta accreta, tubal sterilisation

## Abstract

Although rare, pregnancy can still occur after both tubal sterilization and endometrial ablation. The resulting pregnancy is often complicated by ectopic pregnancy, miscarriage, and adherent placenta. Endometrial ablation is not a contraceptive.

## Graphical abstract

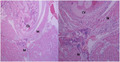

## 1 INTRODUCTION

A case of a 42‐year‐old woman, Para 3 who had an unplanned pregnancy despite bilateral tubal ligation for contraception, and Novasure^®^ endometrial ablation for persistent heavy menstrual bleeding (HMB). The pregnancy was complicated by missed miscarriage at 14 weeks and placenta accreta. This is a rarely reported event.

There is a paucity of data in the literature regarding the incidence of pregnancy after both tubal sterilisation and endometrial ablation. Search of PubMed, Embase, and other online database showed very few case reports such as ours. Among these is the case of pregnancy after hydrothermal endometrial ablation (not Novasure^®^) and laparoscopic sterilization (not postpartum). This resulted in miscarriage at 10 weeks with no adherent placenta recorded. The patient assumed she could not have been pregnant until the 14 weeks of gestation.

Pregnancy should be considered in any woman of reproductive age with irregular bleeding or amenorrhea even after tubal sterilization and endometrial ablation. Women must be counseled that endometrial ablation itself is not a contraceptive procedure. Tubal sterilization is sometimes carried out before or around the same time as endometrial ablation, but women must be counseled that like all other methods of contraception, it may fail.

Postpartum sterilization using the modified Pomeroy technique is an effective method of contraception but with a cumulative failure rate of 7.5/1000 procedures at 10 years.^1^ There are multiple reasons for this failure rate, and effort should be made to reduce operator‐dependent factors. Novasure^®^ is an effective second‐generation endometrial ablation technique for managing HMB but not without risks should subsequent pregnancy occur. Known complications include spontaneous miscarriage, ectopic pregnancy, preterm birth, PAS, and fetomaternal deaths.^2^, ^3^

## 2 CASE HISTORY/EXAMINATION

A case of a 42‐year‐old Para 3, who had all her deliveries by cesarean section. Her last childbirth (LCB) was 7 years prior, during which she underwent bilateral tubal sterilization by modified Pomeroy's technique. The plan for sterilization was made during her antenatal care after counseling and discussion of alternative options. Histology of the specimens confirmed normal Fallopian tubes segments.

Six months later, she suffered HMB which did not respond well to medical treatment including tranexamic acid, mefenamic acid, and oral hormonal treatment. Options, risks, and benefits of further management were discussed with her including Mirena^®^ coil insertion, endometrial ablation, and hysterectomy, with the woman opting for endometrial ablation. One year after her LCB, she underwent Novasure^®^ endometrial ablation. Histological examination of the endometrium revealed no abnormality.

She was amenorrhoeic for 1 year following the ablation, but then developed menometrorrhagia. A planned hysteroscopy to consider Mirena^®^ coil insertion 4 years after endometrial ablation revealed the uterine cavity was obliterated with adhesions. She was not keen on hysterectomy and opted to manage the bleeding irregularity conservatively. There was no evidence of uterine fibroid or adenomyosis from outpatient pelvic ultrasound. She had no other significant medical or surgical history of note. Her cervical smear screening was up to date and normal. She presented to our EPC 7 years after tubal sterilization, and 3 years after endometrial ablation with a positive pregnancy test and uncertain of exact period of amenorrhea.

Her hemoglobin (HB) level from blood test was normal (129 g/L: reference range 115‐165), and blood group B Rhesus D positive.

Transabdominal ultrasound scan showed singleton intrauterine pregnancy with crown‐rump length (CRL) of 81.9 mm corresponding to 14 weeks gestation, with no fetal heartbeat activity. There was Spalding's sign consistent with early fetal demise. The adnexae were normal bilaterally, and a diagnosis of missed miscarriage was made.

Options of management were discussed with her including expectant, medical, and surgical treatment supported with written information. She considered options and decided on medical management. She had two failed cycles of medical treatment of miscarriage with oral mifepristone and misoprostol. SMM was going to be the next management option, and risks and benefits were discussed with her. However, she raised the thought if she could get hysterectomy which has been offered before now for her persistent HMB before she got pregnant. This was discussed at the team level and agreement reached, coupled with the fact that she had completed her family size.

**FIGURE 1:**
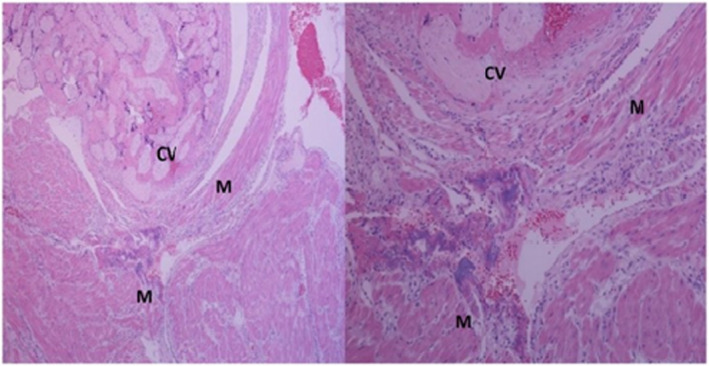
Histologic images showing chorionic villi (CV) in direct contact with the myometrial smooth muscle (M) in keeping with the diagnosis of placenta accreta

She underwent uncomplicated subtotal hysterectomy with an estimated blood loss of 400 milliliters. This procedure was not offered as one of the management options of miscarriage but in view of her peculiar background history and request. Intraoperatively, the Fallopian tubes were noted to be grossly normal‐looking bilaterally, suggesting recanalization of the tubes. She had a good postoperative recovery and was discharged home 2 days after the procedure. Histological examination of the uterus confirmed placenta accreta (Figure 1). She had no concerns at 12 weeks of postoperative follow‐up.

## 3 DISCUSSION

Tubal sterilization is an effective method of contraception in women. Access to the fallopian tube may be via transcervical, laparoscopic, mini‐laparotomy, or during cesarean section (postpartum).^1^ In the United Kingdom (UK), laparoscopic tubal sterilization using clips or rings is the preferred method. Many factors affect sterilization failure, including the experience of the operator, the method and technique used, and characteristics of the patient. These could largely be divided into direct and indirect factors.^4^ A classic example of direct factor would be the timing of the procedure, as in this case the patient had postpartum sterilization which is associated with a higher failure rate and regret.^5^ At 10 years, the failure rate of postpartum partial salpingectomy is 7.5/1000, similar to 8.8/1000 failure rate for postpartum Filshie clip occlusion. Generally, the lifetime failure rate for tubal sterilization is 1/200, while it is 2‐3/1000 procedures at 10 years for laparoscopic tubal occlusion using the Filshie clip.^1^ Patients should be counseled regarding failure rates depending on the timing of the procedure and the method used. The following are some of the documented reasons for tubal sterilization failure:

- Spontaneous recanalization
- Formation of tuboperitoneal fistula
- Selection of wrong anatomical structure (eg, round ligament, peritoneal folds)—This is an example of an operator's error
- Incomplete occlusion of the tube
- Slippage of occlusive device
- Pre‐existing gynecological diseases.^4^, ^6^

It is particularly important for the surgeon to properly identify the tube before and after the procedure to ensure the right structure has been occluded. With partial salpingectomy (Pomeroy's method), histological examination is recommended to confirm complete transection of the tubes, but this does not preclude failure.^7^ We believe tubal sterilization failed in our patient possibly due to spontaneous recanalization as seen during her hysterectomy procedure.

With regard to Novasure^®^ endometrial ablation, it is an effective minimally invasive second‐generation device that uses radiofrequency energy to treat HMB, with success rate of 81%‐90%.^8^ It works by destroying the functional layer of the endometrium; however, because this layer can regenerate, pregnancy is possible afterward. The following are known statistically significant independent risk factors for long‐term Novasure^®^ endometrial ablation failure^8^:

- Younger age group <40 years
- Presence of dysmenorrhea
- Intramural fibroid
- Previous sterilization

It should be noted that these are mainly retrospective studies with conflicting results, especially with sterilization and age group. These studies also showed that previous cesarean section(s) is not associated with an increased rate of Novasure^®^ ablation failure.^8^, ^9^

Generally, the pregnancy rate after ablation is 0.25%‐5.2% depending on the ablative procedure used.^10^ In all, 85% of such pregnancies end as ectopic pregnancy, miscarriage, or termination.^11^ Other complications include preterm labor, intrauterine growth restriction (IUGR), PAS, and perinatal & maternal mortality.^11^, ^12^ Only a few pregnancies, about 1.71%, will be uncomplicated, resulting in term delivery.^13^ These complications are in keeping with our case report where the patient had both spontaneous miscarriage and PAS. Given the destructive nature of the endometrium following radiofrequency ablation, PAS, as seen in our patient, remains a concern and becoming more prevalent. Every postablation pregnancy should be considered to have PAS until proven otherwise, and this was considered in our decision‐making following unsuccessful medical management of miscarriage.^10^, ^11^ One large multi‐institutional cohort study found a PAS rate of 1/13.9 pregnancies after endometrial ablation, in contrast to 1/838 pregnancies in the unexposed group.[14] Another review article quoted a PAS rate of 26% after endometrial ablation.^2^

The rate of amenorrhea at 12 months after Novasure^®^ ablation is 48% to 56%. This does not preclude pregnancy or reintervention later. Our patient had 1‐year amenorrhea postablation but still became pregnant later subsequently. This emphasizes the point that Novasure^®^ is not a contraceptive in itself. This must be highlighted to patients, and ongoing effective contraception strongly advised in women considering endometrial ablation.^14^ It is reported that as much as 80%‐90% of women do not use effective contraception after endometrial ablation.^11^ Our patient already had effective contraceptive in place (tubal sterilization) and assumed she was having early menopause after about 4 months of amenorrhea.

## 4 CONCLUSION

Our case has shown that pregnancy can still happen even after effective contraception following Novasure^®^ endometrial ablation. Endometrial ablation is not suitable for women who are considering pregnancy, or not willing or able to rely on effective contraception after the procedure.^15^, ^16^ Alternative options for management of HMB, including use of Mirena^®^ intrauterine system, should be discussed before any ablative procedure. However, even when all these steps have been taken, pregnancy with notable complications can still occur after endometrial ablation with effective contraception.^15^

## CONFLICT OF INTEREST

No conflict of interest declared.

## AUTHOR CONTRIBUTIONS

NI: wrote the manuscript, did literature review, got patient perspective, and did final editing. KG: suggested the case, did multiple proof reading, and approved the final manuscript. HK: did structural arrangement of the work and grammatical corrections with Grammarly software.

## ETHICAL APPROVAL

No ethical issue of conflict declared. The patient gave an informed written consent for the publication.

## Data Availability

The data used in this manuscript are available online and easily accessible.
